# Neighbourhood active living environment and obesity in adolescents using the Millennium Cohort Study

**DOI:** 10.23889/ijpds.v8i2.2330

**Published:** 2023-09-14

**Authors:** Theodora Pouliou, Lucy Griffiths, Rowena Bailey, Sinead Brophy, Jo Davies, Ronan Lyons, Rebecca Pedrick-Case, Gareth Stratton, Richard Fry

**Affiliations:** 1 ADR Wales, Swansea, United Kingdom; 2 Swansea University, Swansea, United Kingdom

## Objectives

Youth obesity has increased substantially in recent decades; however, the potential role of the built environment in mitigating these trends is unclear. This study examines whether more walkable neighbourhoods are associated with lower levels of overweight/obesity for adolescents compared to less walkable neighbourhoods, after considering the potential role of socio-economic and lifestyle characteristics.

## Methods

We examine overweight/obesity levels for all singleton 14 years-old children living in Wales, using the UK Millennium Cohort Study. Children are classified as healthy weight, overweight and obese using international age and sex adjusted cut-offs for body mass index (BMI). The built environment is assessed using the active living environments (ALE) index for 2017-2018 classified into 5 categories (1-low walkability and 5 – high walkability). We apply regression analysis and adjust for children characteristics (e.g., physical activity), parental socio-economic circumstances and lifestyle choices (e.g., maternal education, physical activity).

## Results

We assess the hypothesis that the built environment is associated with adolescents’ overweight/obesity levels and examine how much of this association could be modified by parental socioeconomic circumstances and lifestyle choices. The ALE index is higher in urban compared to rural areas. To capture variations in Wales’ population, we are conducting a stratified analysis to explore any differences on the association between ALE index and adolescents’ overweight/obesity by urban and rural areas. Accounting for the potential role of lifestyle and socio-economic characteristics is key for future research, as understanding the underlying pathways of this association is necessary to design effective interventions.

## Conclusion

Findings can help us develop a better understanding of associations between the built environment and overweight/obesity status to inform evidence-based planning policy and practice strategies on how to modify the built environment to promote child health in future generations by increasing better opportunities for diet and activity.

